# Disruption of a GATA4/Ankrd1 Signaling Axis in Cardiomyocytes Leads to Sarcomere Disarray: Implications for Anthracycline Cardiomyopathy

**DOI:** 10.1371/journal.pone.0035743

**Published:** 2012-04-20

**Authors:** Billy Chen, Lin Zhong, Sarah F. Roush, Laura Pentassuglia, Xuyang Peng, Susan Samaras, Jeffrey M. Davidson, Douglas B. Sawyer, Chee Chew Lim

**Affiliations:** 1 Molecular Medicine Program, Department of Medicine, Boston University School of Medicine, Boston, Massachusetts, United States of America; 2 Division of Cardiovascular Medicine, Vanderbilt University School of Medicine, Nashville, Tennessee, United States of America; 3 Department of Pathology, Microbiology, and Immunology, Vanderbilt University School of Medicine, Nashville, Tennessee, United States of America; 4 Research Service, Veterans Affairs Tennessee Valley Healthcare System, Nashville, Tennessee, United Sates of America; Istituto Dermopatico dell'Immacolata, Italy

## Abstract

Doxorubicin (Adriamycin) is an effective anti-cancer drug, but its clinical usage is limited by a dose-dependent cardiotoxicity characterized by widespread sarcomere disarray and loss of myofilaments. Cardiac ankyrin repeat protein (CARP, ANKRD1) is a transcriptional regulatory protein that is extremely susceptible to doxorubicin; however, the mechanism(s) of doxorubicin-induced CARP depletion and its specific role in cardiomyocytes have not been completely defined. We report that doxorubicin treatment in cardiomyocytes resulted in inhibition of CARP transcription, depletion of CARP protein levels, inhibition of myofilament gene transcription, and marked sarcomere disarray. Knockdown of CARP with small interfering RNA (siRNA) similarly inhibited myofilament gene transcription and disrupted cardiomyocyte sarcomere structure. Adenoviral overexpression of CARP, however, was unable to rescue the doxorubicin-induced sarcomere disarray phenotype. Doxorubicin also induced depletion of the cardiac transcription factor GATA4 in cardiomyocytes. CARP expression is regulated in part by GATA4, prompting us to examine the relationship between GATA4 and CARP in cardiomyocytes. We show in co-transfection experiments that GATA4 operates upstream of CARP by activating the proximal CARP promoter. GATA4-siRNA knockdown in cardiomyocytes inhibited CARP expression and myofilament gene transcription, and induced extensive sarcomere disarray. Adenoviral overexpression of GATA4 (AdV-GATA4) in cardiomyocytes prior to doxorubicin exposure maintained GATA4 levels, modestly restored CARP levels, and attenuated sarcomere disarray. Interestingly, siRNA-mediated depletion of CARP completely abolished the Adv-GATA4 rescue of the doxorubicin-induced sarcomere phenotype. These data demonstrate co-dependent roles for GATA4 and CARP in regulating sarcomere gene expression and maintaining sarcomeric organization in cardiomyocytes in culture. The data further suggests that concurrent depletion of GATA4 and CARP in cardiomyocytes by doxorubicin contributes in large part to myofibrillar disarray and the overall pathophysiology of anthracycline cardiomyopathy.

## Introduction

Adriamycin (doxorubicin) is an effective anti-cancer drug, whose use is limited by the development of a dose-dependent cardiomyopathy and congestive heart failure. Cardiac tissue from animals and patients treated with doxorubicin are histologically characterized by swelling of the sarcoplasmic reticulum and mitochondria, cytoplasmic vacuolization, and widespread loss and disarray of sarcomeres (for reviews see [1], [2]). Cardiac sarcomeres are highly organized structures and maintain a strict stoichiometry of myofilament proteins allowing efficient generation of contractile force [3]. Myofilament stoichiometry, in turn, relies on the coordinated turnover of myofilament proteins that efficiently replaces worn out or damaged myofilament proteins. This equilibrium is presumably regulated by sarcomeric elements able to mechanically “sense” myofilament protein deficits and signals the cardiomyocyte to induce myofilament gene transcription.

Cardiac ankyrin repeat protein (CARP, a.k.a. cardiac adriamycin responsive protein and ANKRD1) is a member of a family of conserved muscle ankyrin repeat proteins (MARPS) that include ankrd2 and diabetes ankyrin repeat protein (DARP) [4], [5], [6]. CARP was originally discovered as the nuclear protein C-193 [7] but later independently characterized by Zou et al. as a co-factor for transcription factor YB-1 and by Jeyaseelan et al. as a gene whose mRNA was “exquisitely sensitive” to doxorubicin treatment [8], [9]. Due to its association with the transcriptional repressor YB-1, CARP was originally thought to act as a suppressor of cardiac genes, including myosin light chain 2v (MLC-2v), atrial natriuretic factor (ANF), and cardiac troponin C (cTnC). In three distinct models of cardiac hypertrophy in rats (constriction of abdominal aorta; spontaneously hypertensive; Dahl salt-sensitive) Aihara et al. found increased CARP expression [10]. In addition to YB1, CARP has been shown to interact with sarcomeric proteins: myopalladin, desmin, muscle specific RING finger proteins (MuRFs), the N2A portion of titin, cardiac calsequestrin and CARP itself [11], [12], [13], [14]. In cardiomyocytes in culture, CARP has been shown to be essential for sarcomere organization through its interaction with the sarcomere protein myopalladin [11]. Recently, several missense mutations in the CARP gene, ANKRD1, were identified in patients with dilated and hypertrophic cardiomyopathy [15], [16], and *in vitro* studies of these mutations suggest disruption of CARP localization and cardiac stretch-based signaling. Given its dual subcellular localization, within the nucleus and sarcomere, it has been proposed that CARP is part of a sarcomeric complex, capable of sensing and relaying a muscle stretch signal to induce gene expression [6], [17].

GATA4 (a member of the GATA family of zinc finger transcription factors) is expressed in the adult heart and is important in regulating cardiac hypertrophy and cardiomyocyte survival [18]. Previous studies in cardiomyocytes have shown that doxorubicin depletes GATA4 levels and induces apoptosis and that restoration of GATA4 levels by adenovirus mediated gene transfer is able to prevent the doxorubicin-induced autophagy and apoptosis [19], [20], [21]. Interestingly, CARP has been shown to be a downstream target of GATA4 [22], [23]. Thus GATA4 and CARP could be part of a signaling pathway that is important for sarcomere organization and cardiomyocyte survival.

In this study we explored a possible link between the disruption of GATA4/CARP signaling and doxorubicin-induced sarcomere disarray in rat cardiomyocytes. Our results demonstrate that both CARP and GATA4 maintain sarcomere integrity by regulating myofilament gene transcription, and that loss of either CARP or GATA4 *in vitro* directly contributes to myofibrillar disarray. These observations offer insight into the role of GATA4 and CARP in sarcomere homeostasis and may lead to novel therapeutic strategies that can treat or limit the debilitating effects of doxorubicin cardiomyopathy.

## Materials and Methods

### Ethics Statement

This study was carried out in accordance with the recommendations of the Guide for the Care and Use of Laboratory Animals of the National Institutes of Health. The protocols for all experiments using vertebrate animals were approved by the Institutional Animal Care and Use Committee at Vanderbilt University Medical Center.

### Primary Isolation of Cardiac Myocytes and Cell Culture

Adult rat ventricular myocytes (ARVMs) were isolated from male Sprague-Dawley rats (∼200 g), plated on laminin-coated culture dishes and cultured, as previously described [24]. Culture medium contained Dulbecco's modified Eagle's medium (DMEM, Invitrogen) supplemented with 7% fetal bovine serum (FBS, Invitrogen), 2 mg/mL albumin (Sigma), 5 mM creatine (Sigma), 2 mM L-carnitine (Sigma), 5 mM taurine (Sigma), 1% 100 units/ml penicillin/streptomycin (Invitrogen), and 100 µM bromodeoxyuridine (BrdU, Sigma). Culture media was changed 1 h after plating and every two days until the day of the experiment. Myocytes were treated on day 9 or 10 in culture with: doxorubicin (Sigma), N-acetyl-L-leucyl-norleucinal (ALLN, Sigma), cycloheximide (Sigma), actinomycin D (Sigma) at concentrations and time-points indicated elsewhere.

Neonatal rat ventricular myocytes (NRVMs) were isolated from hearts of 2-day old Sprague-Dawley rat pups as previously described [24], and cells were cultured in DMEM supplemented with 7% FBS. NRVMs were cultured overnight in low serum media (1% FBS/DMEM) prior to plasmid transfection, adenoviral infection at a multiplicity of infection of 100 (MOI 100), and/or chemical treatment.

### Quantitative Real-Time RT-PCR

Total RNA from isolated ARVMs was extracted using TRIzol reagent (Invitrogen) using standard methods. One step quantitative real-time RT-PCR was performed using the QuantiTect SYBR Green kit from Qiagen with a Cepheid SmartCycler. Primers were designed to a region of the rat CARP gene sequence spanning exons 3–5 (forward: 5′-GAAGGAACCGGAGCCTGA-3′; reverse: 5′-CACGATCGCC AAGTGTCC-3′). Samples were normalized by real-time RT-PCR with an internal control using primers targeting the 18S ribosome protein L32 (forward 5′-ACTGGAATTCGCTGCCCTCCGGCCT-3′; reverse: 5′-GCATAAGCTTTCGGTCTGA CTGGTG-3′. Standard curves were generated to determine the linear range and efficiency for each primer set. Experiments were repeated in triplicate and C_T_ values were measured 5 times per individual sample. The high and low values were removed and the remaining 3 were averaged.

### Constructs and siRNAs

We generated promoter reporter constructs by cloning from mouse cDNA a putative CARP promoter region (−660 to 0 bp) and from human cDNA the cardiac alpha actin promoter region (−557 to 0 bp) and titin promoter region (−548 to 0 bp) into the pGL3 luciferase reporter plasmid (Promega). The FLAG-tagged GATA4 expression vector was a kind gift from Dr. Mona Nemer. An adenovirus containing full-length mouse CARP cDNA was generated as previously described [25]. We obtained a truncated N-terminal epitope of myomesin fused to GFP (kind gift from Dr. Jeane-Claude Perriard) and cloned it into a tet-regulatable adenoviral construct. The CMV-EGFP adenovirus (Cell Biolabs, Inc) and the CMV-GATA4 adenovirus (Seven Hills Bioreagents) were purchased. All adenoviruses were amplified and purified to high titer (Virabind, Cell Biolabs, Inc).

Short interfering RNA (siRNA) were designed and synthesized against exon 4 of the rat CARP sequence (NM_0.1220.1, CARP-siRNA: 5′-GAACCGGAGCCTGAAATTATT-3′), rat GATA4 sequence (NM_144730, predesigned siRNA from Qiagen), and siRNA non-silencing control (5′-AATTCTCCGAACGTGTCACGT-3′). Lipofectamine 2000 (Invitrogen) and siRNA were diluted with Opti-MEM (Invitrogen) and the mixture was subsequently transfected into ARVMs or NRVMs. After 4 h, the media was changed to DMEM supplemented with 7% FBS, penicillin-streptomycin and BrdU under normal growth conditions. Cells were lysed for analysis up to 96 h after transfection.

### Gel electrophoresis and immunoblotting

Cells were lysed in modified RIPA buffer: 10% NP-40, 0.5 M EDTA, 0.5 M Tris-HCl (pH 7.4), 5 M NaCl, 1 M NaF, protease inhibitor cocktail (P8340, Sigma) and PMSF (1∶100). The samples were centrifuged at 14,000 rpm at 4°C to pellet out cellular debris. The supernatant was measured for protein concentration using the Bio-Rad RC/DC protein assay. Cell lysates were subsequently boiled in Laemmli sample buffer, loaded, and separated on 12% Tris-HCl polyacrylamide gels (Cambrex). The gel was transferred to PVDF membranes (Amersham Biosciences) for immunoblotting.

The affinity purified CARP polyclonal rabbit antibody was produced from a peptide encoded by an N-terminal 300-bp fragment of CARP cDNA (bases 31–349), as previously described [25]. Membrane blots were incubated with antibodies to either CARP (diluted in 5% BSA/0.1% TBST), GATA4 (Santa Cruz Biotech, clone G4, sc-25310) total actin (Sigma, A-3853, clone AC-40) or α-tubulin (Santa Cruz Biotech, sc-5286, clone B-7) overnight at 4°C, followed by anti-rabbit or anti-mouse IgG conjugated to HRP (Santa Cruz Biotech,) for 1 h at room temperature. All primary antibodies were used at 1∶1,000 dilution and secondary antibody at 1∶5,000. Reactive bands were detected on film by chemiluminescence (Pierce Chemical Co.), scanned, and band densities were quantitated using ImageJ (NIH) software.

For titin analysis, cells were lysed in a modified Laemmli buffer according to Warren et al. [26], with the addition of protease inhibitors (0.04 mM Leupeptin, 0.01 mM E-64, and 0.5 mM PMSF). Samples were heated at 60°C and immediately loaded on 1% agarose gels. Gels were fixed and following Coomassie staining, wet-scanned at 400 dpi.

### Immunofluorescence

Cardiomyocytes were fixed with 4% paraformaldehyde/PBS for 30 minutes, and permeabilized with 0.2% Triton-X/PBS at 30 minutes, as previously described [24]. Samples were incubated with primary antibodies to myomesin (B4, University of Iowa Hybridoma Bank), α-actinin (Santa Cruz Biotech), or CARP, followed by incubation with fluorophore-conjugated secondary antibodies (Alexa dyes, Invitrogen) and Texas-Red phalloidin (for staining filamentous actin). The cells were mounted on a coverslip using VectaShield (Vector Labs) mounting media and sarcomere morphology was analyzed using an inverted fluorescence confocal microscope (LSM 510, Zeiss). To quantify myofibrillar damage, myocytes were score based on >50% disruption of myomesin striations, accumulation of dense myomesin aggregates, or reduction in actin staining as previously described [24]. For each condition in each experiment, a total of 100–150 cardiomyocytes were counted by an experimenter blinded to the experiment.

### Myomesin-GFP Transfection and Time-lapse Fluorescence Microscopy

ARVMs were infected with myomesin-GFP and Tet-off adenoviral vectors at an MOI of 1 and at ratio of 1∶1. Cells were incubated for approximately 24 h at which time myomesin-GFP fluorescence was visible. The media was replaced with phenol-free media and the fluorescent cells were transfected with CARP siRNA-325 or non-silencing siRNA and allowed to incubate up to 4 days.

Time-lapse microscopy was conducted using an Olympus IX81 inverted fluorescent microscope outfitted with a PrecisionControl WeatherStation chamber for maintaining CO_2_, temperature and humidity levels. Cells were subjected to time-lapse (Slidebook software, Intelligent Imaging Innovations, Denver, CO) and images were recorded on a single field excited at 489 nm at 1, 4, and 6 days.

### Luciferase-Reporter Assay

The luciferase-reporter transfections were carried out in NRVMs cultured on 24-well plates at 70–80% confluence using Lipofectamine 2000 (Invitrogen) following manufacturer's instructions. NRVMs were transfected with 200 ng of the promoter luciferase construct for 24 h. Transfection efficiency was monitored by co-transfecting NRVMs with 200 ng pRL-TK vector (Promega) that contains the renilla luciferase gene. Firefly and renilla luciferase activities were determined by a GloMax-Multi Detection system (Promega). The ratio of firefly∶renilla luciferase activities was calculated to normalize for differences in cell number and transfection efficiency. Each experiment was run in triplicate and the ratio value was determined for each construct to allow for between-experiment comparison.

### Statistical Analysis

Data are reported as mean±SD. Where appropriate, results were either analyzed by Student's t-test or ANOVA with a Bonferroni's multiple comparison post-hoc test. *P*<0.05 was considered statistically significant.

## Results

### CARP expression and localization in Adult Rat Ventricular Myocytes (ARVM)

**Figure 1:**
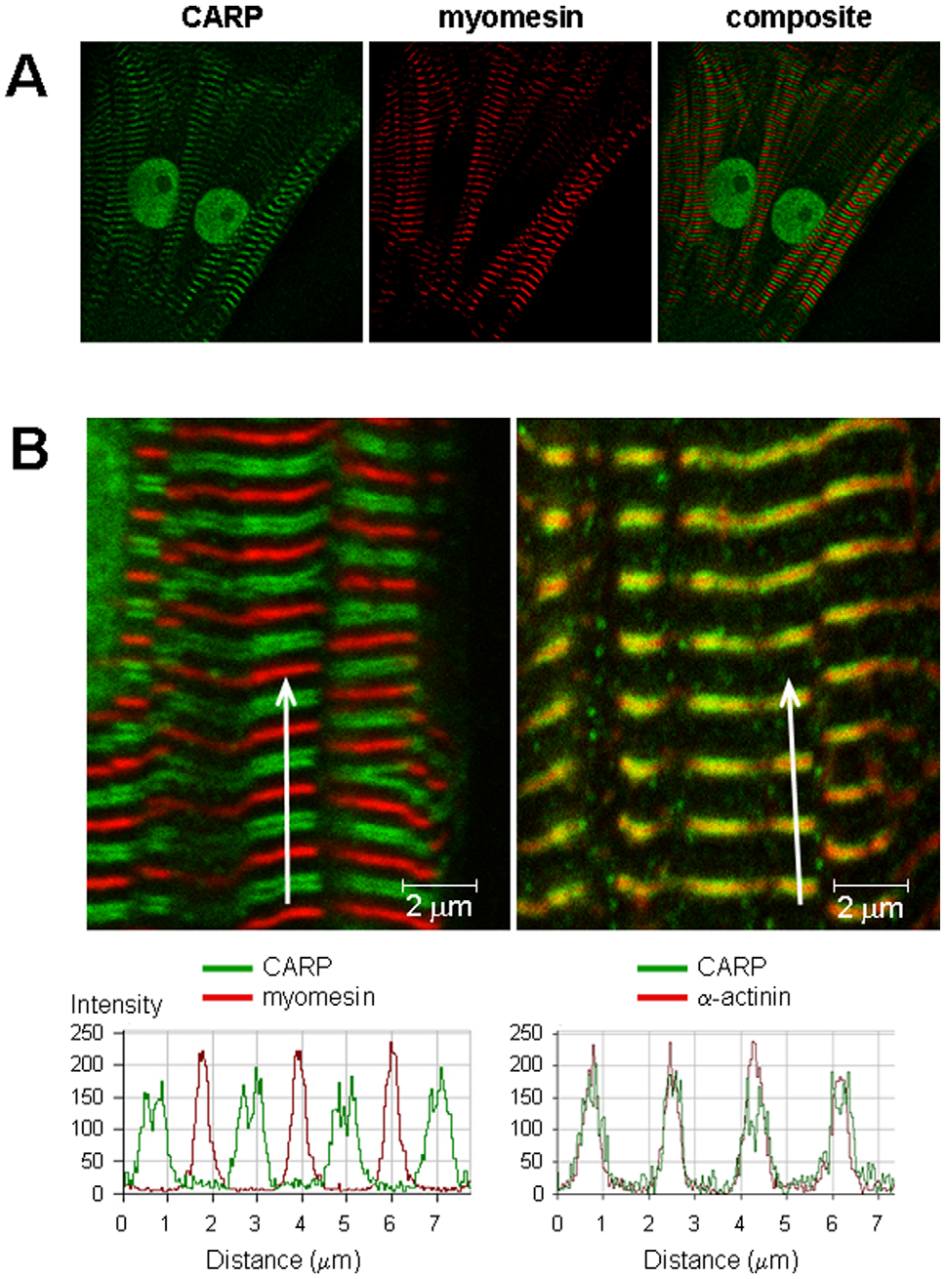
Sarcomeric and nuclear localization of CARP in ARVMs. **A:** Low magnification (40×) images of ARVMs co-stained with antibodies to CARP (green) and myomesin (red). **B:** High magnification (100×) of ARVMs immunostained for CARP (green) and myomesin (red, left image) or α-actinin (red, right image), and shown below the image is the corresponding fluorescence intensity along the white arrow in the red and green channels.

We used the primary dedifferentiated adult rat ventricular cardiomyocyte (ARVM) model and were able to confirm CARP mRNA and protein expression in these cells (supplemental, **Figure S1**). Immunostaining of CARP in paraformaldehyde-fixed ARVMs showed a striated sarcomeric pattern with intense staining in the nucleus (**Figure 1A**). Confocal images taken from ARVMs co-immunostained with CARP and either α-actinin (Z-line protein) or myomesin (M-line protein) revealed CARP staining adjacent to the Z-line (**Figure 1B** **, Figure S2**), which corresponds to CARP localization at the I-band. This is in agreement with previous studies in mouse ventricular tissue and neonatal rat and embryonic chick cardiomyocytes, showing that CARP localized in the nucleus and at the N2A region of titin in the I-band [6], [11].

### CARP is susceptible to doxorubicin treatment

**Figure 2:**
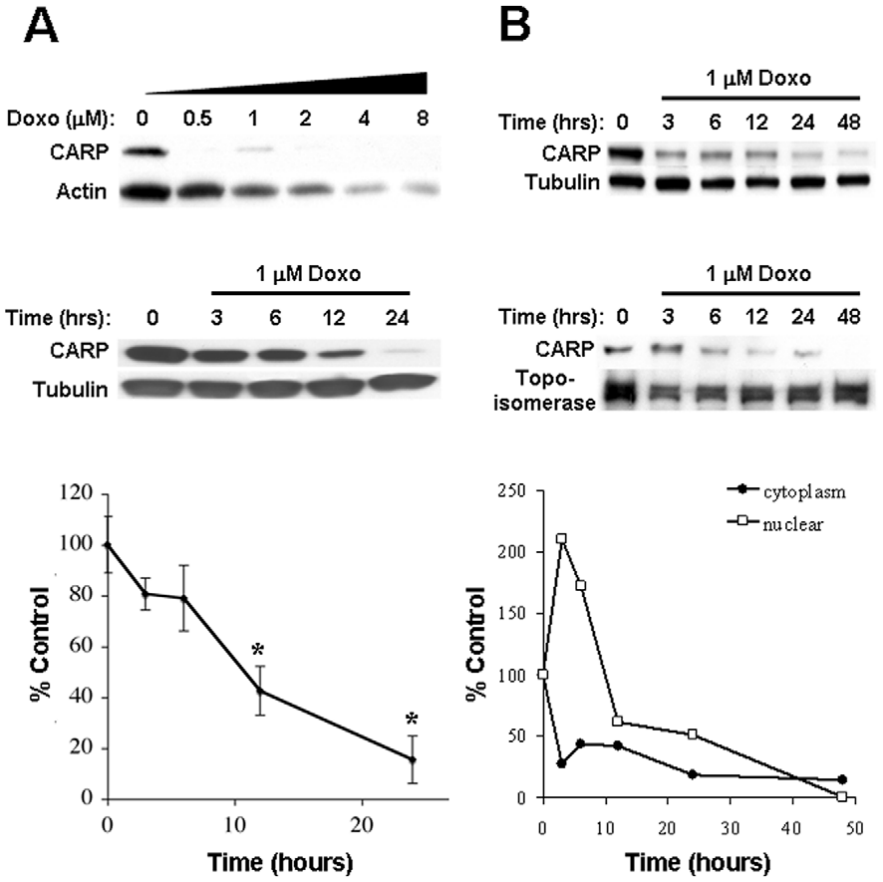
Doxorubicin decreases CARP levels in ARVMs. **A:** ARVMs were treated for 24 h with increasing concentrations of doxorubicin and cell lysates analyzed by immunoblot for CARP and actin. Also shown is a time course of CARP and tubulin expression in ARVMs treated with 1 µM doxorubicin. CARP densitometry values for the time-course were normalized to tubulin and expressed as a percentage of the 0 time-point. Shown are mean±SD from 4 independent experiments. * *P*<0.05 relative to 0 time-point. **B:** Cells were harvested and fractionated at various time points into nuclear and cytoplasmic extracts and analyzed for CARP expression by immunoblot. CARP densitometry values were normalized to tubulin (cytoplasmic fraction) or topoisomerase (nuclear fraction) and expressed as a percentage of the 0 time-point.

**Figure 3:**
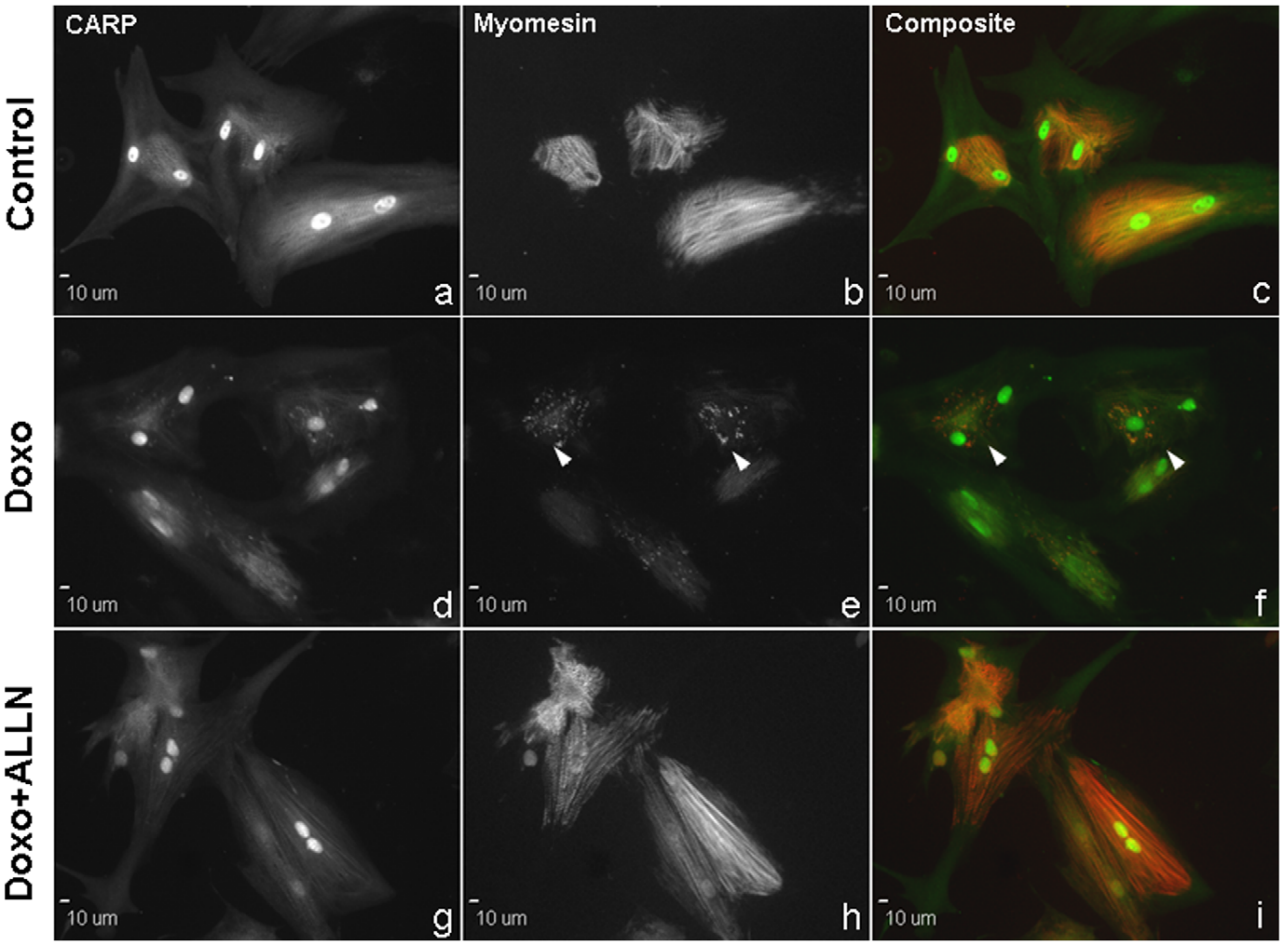
Calpain inhibition preserves sarcomere structure but not CARP levels in ARVMs treated with doxorubicin. **A:** Immunofluorescent images of untreated ARVMs (a–c) or ARVMs treated for 24 h with 1 µM doxorubicin (Doxo, d–f) in the presence or absence of 100 µM N-acetyl-L-leucyl-norleucinal (ALLN, g–i). ARVMs were co-stained for CARP (green) and myomesin (red); arrowheads indicate presence of dense myomesin aggregates.

To determine the susceptibility of CARP to doxorubicin, ARVMs were incubated at different concentrations of doxorubicin for 24 h and CARP levels analyzed by immunoblot. Our data show that CARP is extremely sensitive to doxorubicin when compared to actin; as little as 0.5 µM doxorubicin was sufficient to suppress CARP protein expression and a time course of ARVMs treated with 1 µM doxorubicin showed a significant decrease in CARP levels as early as 3 h to less than 20% of control by 24 h (**Figure 2A**). The immunoblot data were corroborated by immunofluorescent images of ARVMs treated for 24 h with 1 µM doxorubicin, with immunostaining of sarcomeric and nuclear CARP often appearing diffuse and less intense compared to control cells (**Figure 3**). Since CARP resides in both the cytoplasm and nucleus of cardiomyocytes, we examined the relative susceptibility of CARP to doxorubicin in both compartments. Interestingly, there was a decrease in cytoplasmic CARP concomitant with a transient increase in nuclear CARP followed by an overall decrease in CARP to <20% by 48 h (**Figure 2B**). This was confirmed by immunostaining of CARP in doxorubicin treated ARVMs, which showed increased staining of nuclear CARP at 6 and 12 h followed by an overall decrease in CARP immunostaining at later time-points (**Figure S3**).

### Calpain inhibition prevents myofibrillar disarray and preserves titin but not CARP levels

**Figure 4:**
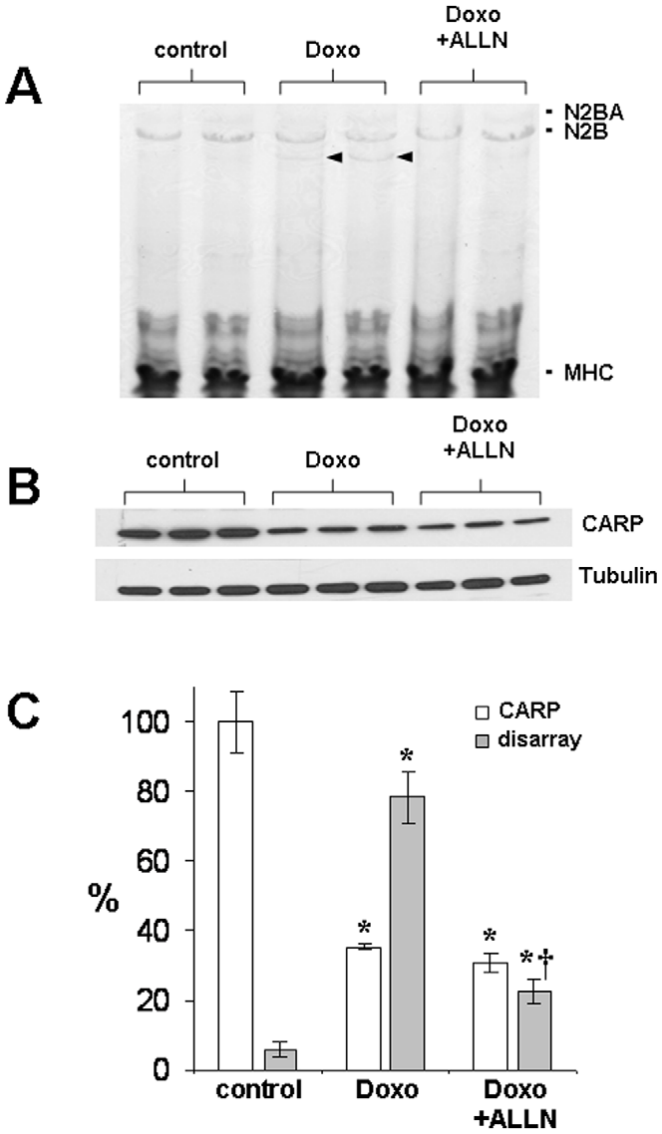
Calpain inhibition preserves titin but not CARP levels. ARVMs were treated for 24 h with 1 µM doxorubicin and cell lysates analyzed for **A:** titin in Coomassie stained agarose gels (representative of 4 independent experiments, arrowheads indicate titin degradation product), and **B:** CARP and tubulin by immunoblot. **C:** Bar graph shows corresponding quantification of CARP immunoblot analysis (% of control, n = 3) and myofibrillar disarray (% of cells scored for >50% disruption of myomesin striations as described in Methods, n = 4–5, ∼150 cells counted per experiment). Shown are mean±SD, *P*<0.05 relative to control (*) and Doxo (**†**).

We have previously shown that doxorubicin induces titin degradation in ARVMs and that co-treatment with N-acetyl-L-leucyl-norleucinal (ALLN), an inhibitor of the calcium-dependent calpain proteases, was able to preserve titin levels and prevent myofibrillar disarray [24]. Given that CARP interacts with I-band titin, it is also possible that calpain-induced proteolysis of titin releases bound CARP, making it more susceptible to degradation. Thus, we determined whether preservation of titin levels by calpain inhibition during doxorubicin treatment could also stabilize and preserve CARP levels. We examined the effects of doxorubicin on titin by agarose gel electrophoresis and sarcomere structure by evaluation of the immunostaining pattern of myomesin [24], [27]. ARVMs treated with 1 µM doxorubicin for 24 h showed partial degradation of titin (**Figure 4A**) as previously reported [24], [27], decreased CARP levels, and significant myofibrillar disarray with dense aggregation and reduced staining of myomesin (**Figures 3** **,** **4B, and 4C**). Pretreating ARVMs with 100 µM ALLN prior to doxorubicin preserved titin levels and almost completely restored myomesin staining, but ALLN did not restore either CARP protein levels or its staining pattern (**Figures 3** **and** **4**).

These data suggest that CARP degradation is not mediated by the calpains and that preservation of titin and/or myofilament structure during doxorubicin treatment does not preserve sarcomeric localization or the stability of CARP. Hence titin degradation is not a prerequisite for doxorubicin-induced degradation of CARP.

### Doxorubicin suppresses CARP through a transcriptional mechanism

**Figure 5:**
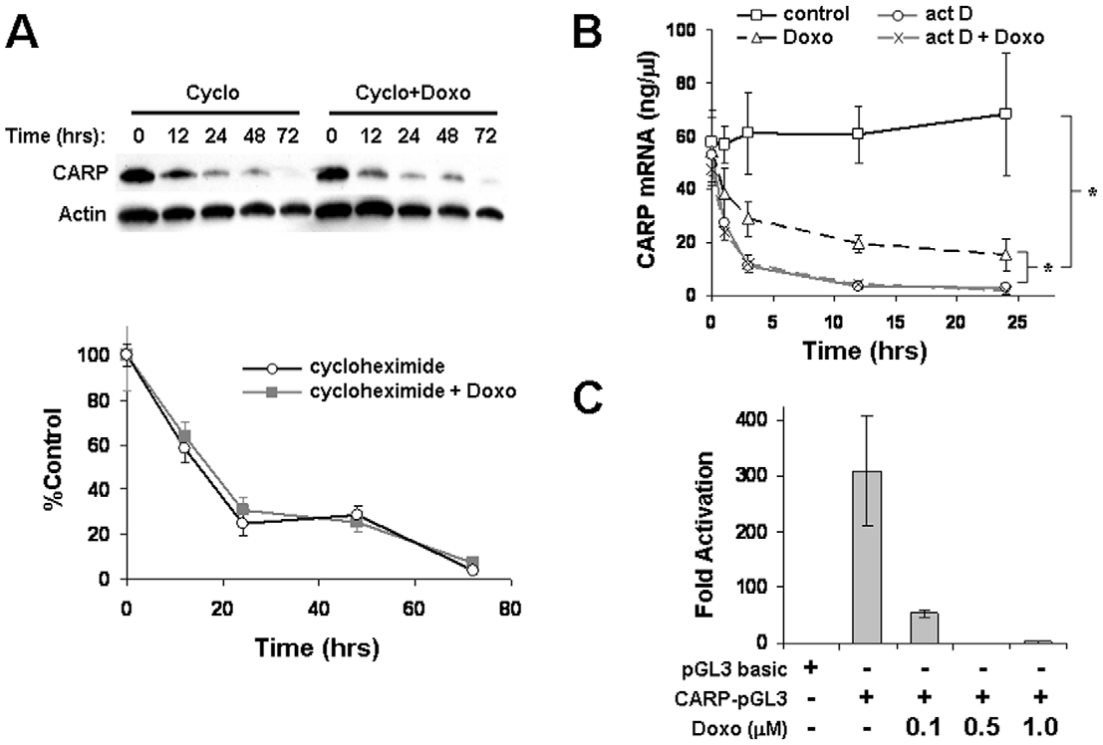
Doxorubicin inhibits CARP expression at the transcriptional level. **A:** ARVMs were pretreated with 10 µg/ml cycloheximide (Cyclo), a protein synthesis inhibitor, in the presence or absence of 1 µM doxorubicin (Doxo) and cell lysates analyzed by immunoblot for CARP and actin and corresponding densitometry analysis is shown below. **B:** Comparison of CARP mRNA decay (quantified by RT-PCR) in ARVMs pretreated with 5 µg/ml actinomycin D (act D) in the presence or absence of 1 µM doxorubicin. **C:** NRVMs were transfected with a CARP promoter luciferase reporter (CARP-pGL3) and treated with increasing concentrations of doxorubicin. Cell lysates were assayed for luciferase activity and values were normalized to a promoterless control (pGL3 basic). Shown are mean±SD from 4 independent experiments. The qRT-PCR and luciferase-reporter experiments were performed in triplicate. * *P*<0.05, ANOVA.

Since doxorubicin-induced CARP reduction was calpain-independent, we examined whether doxorubicin enhanced degradation of CARP protein by any other proteolytic system. ARVMs were pretreated with 10 µg/ml cycloheximide, a protein biosynthesis inhibitor, for 1 h prior to treatment with 1 µM doxorubicin (the control group was treated only with cycloheximide). ARVMs were harvested over various time-points to compare the rate of CARP degradation between the two groups. Western blot analysis showed no significant difference in the rate of CARP degradation between cycloheximide alone and in combination with doxorubicin (**Figure 5A**). Similarly, we wanted to ascertain whether doxorubicin inhibits CARP transcription or destabilizes CARP mRNA. ARVMs were pretreated for 1 h with 5 µg/ml actinomycin D, a transcriptional inhibitor, prior to treatment with 1 µM doxorubicin, and cells were lysed over various time points for total RNA extraction. We also treated cells individually with either actinomycin D or with doxorubicin. By quantitative RT-PCR analysis, ARVMs treated with doxorubicin alone showed a rapid decline in CARP mRNA, whereas untreated control demonstrated stable levels of CARP mRNA (**Figure 5B**). However, ARVMs treated with actinomycin D alone versus actinomycin D with doxorubicin had an equivalent rate of CARP mRNA reduction, suggesting that CARP mRNA degradation is not enhanced with doxorubicin.

To confirm that doxorubicin inhibits CARP at the transcriptional level, neonatal rat ventricular myocytes (NRVM) were transfected with a CARP promoter-luciferase reporter (CARP-pGL3) and treated with different concentrations of doxorubicin. The CARP promoter was strongly activated under basal conditions (∼300 fold) in NRVMs; doxorubicin induced a dose-dependent decrease, with complete suppression of CARP promoter activity at 0.5 and 1.0 µM doxorubicin (**Figure 5C**).

These findings suggest that the decrease in CARP expression during doxorubicin treatment was neither due to enhanced CARP protein degradation nor destabilization of mRNA transcripts, but rather due to suppression of CARP mRNA transcription.

### CARP siRNA induces myofibrillar disarray

**Figure 6:**
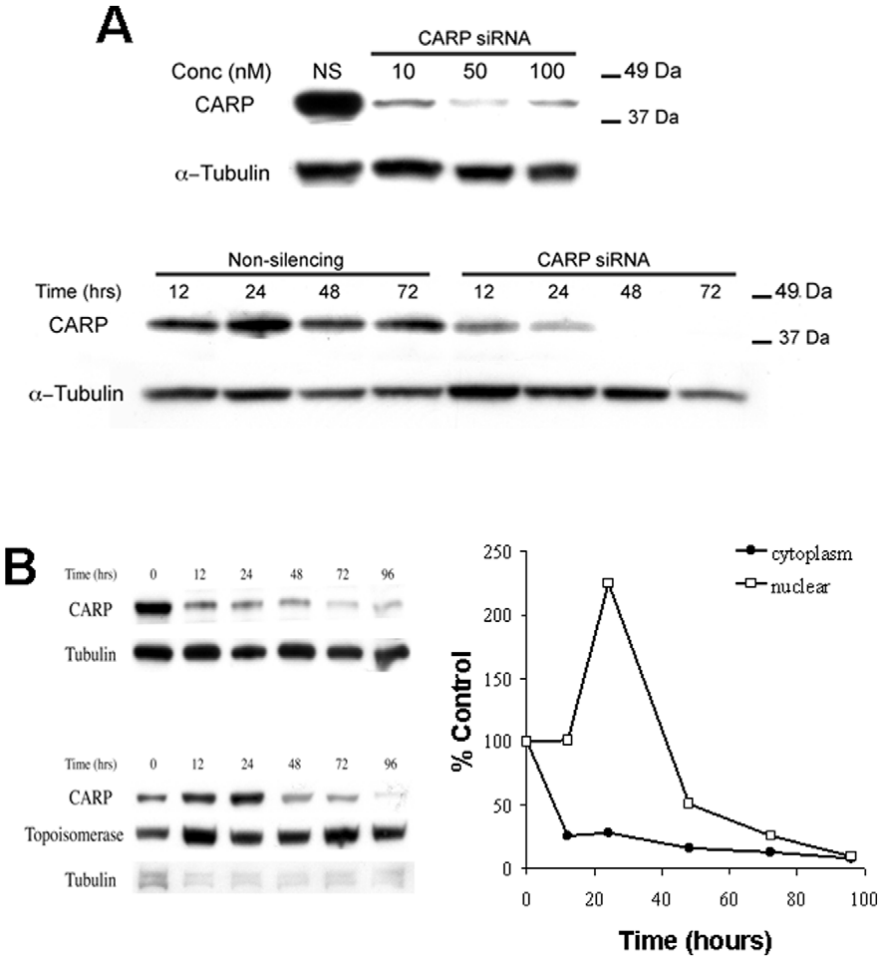
CARP siRNA knockdown in ARVMs. **A:** ARVMs were transfected for 24 h with increasing concentrations of CARP targeted siRNA and lysates analyzed by immunoblot for CARP and tubulin. Also shown is a time course of CARP and tubulin levels in ARVMs transfected with 50 nM CARP- siRNA. **B:** Cells were harvested at various time-points and fractionated into nuclear and cytoplasmic extracts and analyzed for CARP expression by immunoblot. CARP densitometry values were normalized to tubulin or topoisomerase and expressed as a percentage of the 0 time-point.

**Figure 7:**
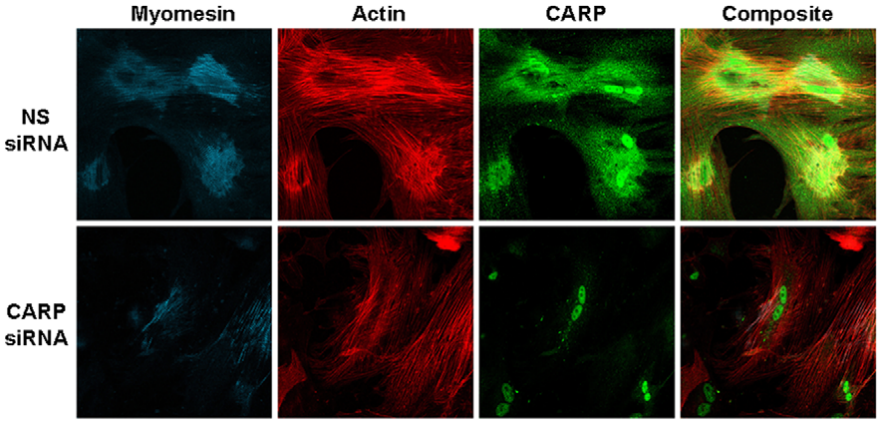
CARP knockdown in ARVMs induces sarcomere disarray. **A:** ARVMs were transfected with 50 nM nonsilencing siRNA (top panel) or 50 nM CARP-siRNA (bottom panel and fixed at 48 h for immunofluorescence imaging. Cells were stained for CARP (green) myomesin (blue), and filamentous actin (red).

Doxorubicin has well-known pleiotropic effects. To better define the consequences of CARP inhibition alone, we utilized siRNA to specifically knockdown CARP levels. CARP-siRNA suppressed CARP mRNA transcripts by over 75% compared to a non-silencing siRNA transfected control. A dose response and time-course revealed almost complete suppression of CARP protein by 48 h in ARVMs treated with 50 nM CARP siRNA (**Figure 6A**). CARP-siRNA had no effect on GATA4 and phospho-ERK1/2 levels suggesting no or minimal off-target effects (data not shown). Immunofluorescence studies revealed that ARVMs transfected with 50 nM CARP siRNA for 48 h had significantly reduced sarcomeric CARP immunostaining, with some retention in the nuclei, and this was accompanied by drastic decreases in filamentous actin and myomesin striations within the cell (**Figure 7**). Quantification of myofibrillar damage showed a significant increase in sarcomere disarray in CARP-siRNA treated ARVMs (55.0±10.6% vs. 6.8±0.6%, n = 3, ∼150 cells counted per experiment, * *P*<0.05 relative to control). Analysis of the effect of CARP siRNA on the cytoplasmic and nuclear CARP compartments revealed similar dynamics in CARP distribution as seen with doxorubicin, however, the transient increase in nuclear CARP occurred much later in CARP-siRNA compared to doxorubicin treated cells (compare **Figures 2B** and **6B**, and **Figure S3**).

To visualize sarcomere changes due to CARP silencing in real-time, ARVMs were infected with an adenovirus expressing a truncated myomesin-GFP (which localizes to the M-line). After 24 h cells were treated with CARP-siRNA and changes in myomesin-GFP were tracked using time-lapse fluorescence microscopy. Sarcomeric M-line striations were visible 24 h after infection and would persist at 4 days in untreated and non-silencing siRNA controls (**Figure S4**). CARP suppression resulted in a gradual loss of M-line striations by day 2 along with accumulation of densely fluorescent intracellular aggregates. Thus, targeted suppression of CARP recapitulates the doxorubicin-induced sarcomeric injury phenotype.

### CARP overexpression does not rescue doxorubicin-induced sarcomere disarray

**Figure 8:**
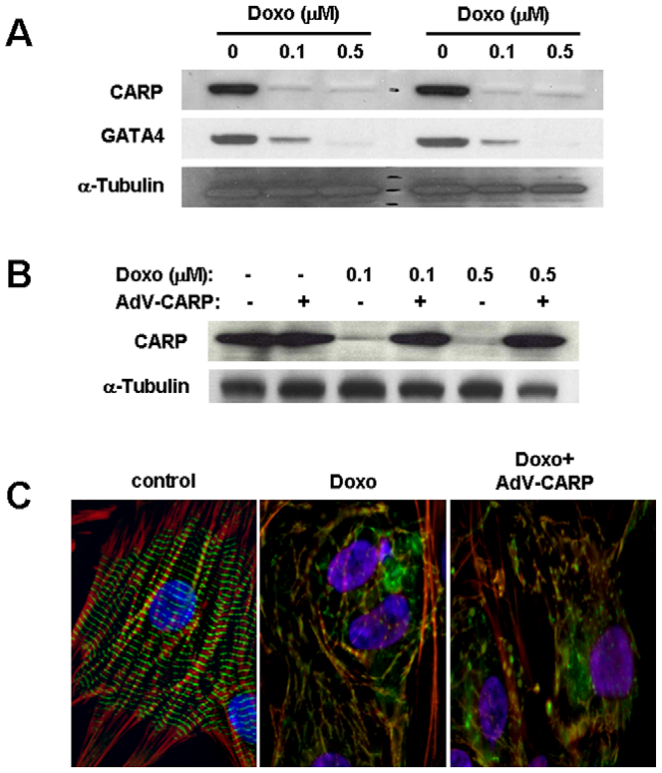
Preservation of CARP in NRVM does not rescue doxorubicin-induced sarcomere disarray. **A:** NRVMs were treated for 24 h with increasing concentrations of doxorubicin and cell lysates analyzed by immunoblot for CARP, GATA4, and tubulin. **B:** NRVMs were treated with 0.5 µM doxorubicin alone or infected with adenoviral CARP (AdV-CARP) for 24 h followed by doxorubicin treatment for 24 h and cell lysates were analyzed by immunoblot for CARP and tubulin. **C:** Immunofluorescent images of untreated control, NRVMs treated with 0.5 µM doxorubicin alone or NRVM infected with AdV-CARP for 24 h followed by doxorubicin for 24 h. NRVMs were co-stained for myomesin (green) and filamentous actin (red). Note: the purple appearance of nuclei is due to the overlay of DAPI staining with red autofluorescence from doxorubicin intercalated into nuclear DNA.

Since CARP-siRNA knockdown in cardiomyocytes induces a similar sarcomere disarray phenotype as seen with doxorubicin, we sought to determine if CARP overexpression can rescue doxorubicin-induced sarcomere disarray. We used adenovirus-mediated gene transfer to overexpress CARP (AdV-CARP) in cardiomyocytes. NRVMs treated with 0.1 or 0.5 µM doxorubicin for 24 h resulted in marked depletion of CARP (**Figure 8A**). NRVMs pre-infected with AdV-CARP for 24 h followed by 0.1 or 0.5 µM doxorubicin maintained their CARP similar to control levels (**Figure 8B**). However, this preservation of CARP failed to rescue the doxorubicin-induced sarcomere disarray phenotype, as shown by the aberrant M-line immunostaining (**Figure 8C**); quantification of sarcomere disarray (Dox = 69.9±5.7%, vs. Dox+AdV-CARP = 66.5±8.0%, n = 4, ∼150 cells counted per experiment, *P* = NS). Thus, CARP depletion alone does not account for the doxorubicin-induced sarcomere disarray phenotype.

### GATA4 is an upstream regulator of CARP

**Figure 9:**
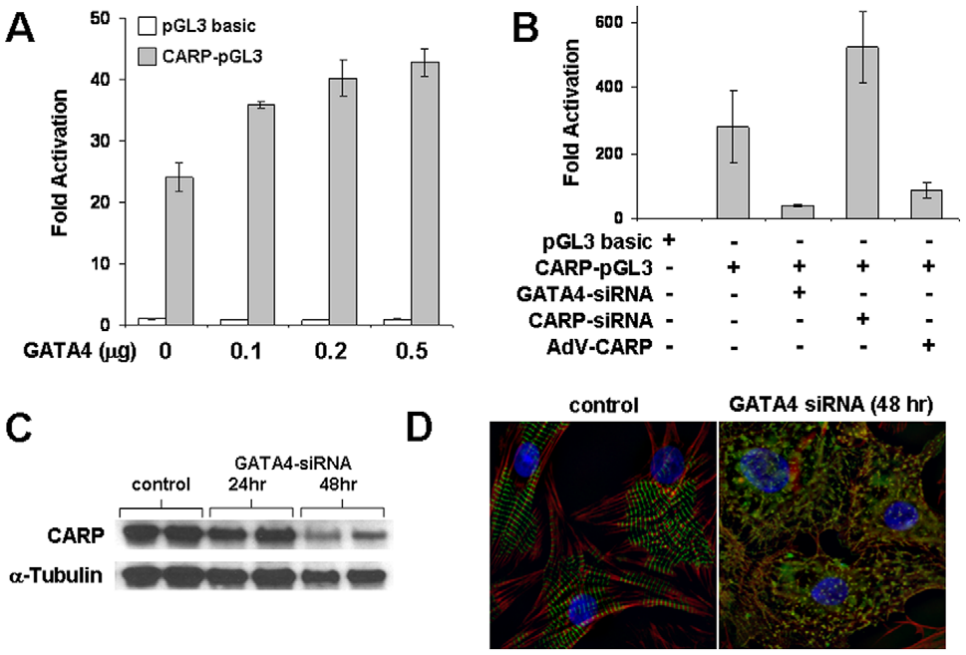
GATA4 is an upstream regulator of CARP. **A:** HEK-293 cells were cotransfected with CARP-pGL3 promoter (filled bars) and increasing concentrations of GATA4 expression plasmids and cell lysates were assayed for luciferase activity. The experiment was repeated with a promoterless pGL3 vector (open bars) as control. **B:** NRVMs were transfected with CARP-pGL3 along with either GATA4-siRNA, CARP-siRNA, or AdV-CARP for 24 h and cell lysates were assayed for luciferase activity. All luciferase-reporter experiments were performed in triplicate. Values were normalized to untreated pGL3 basic and shown as mean±SD from 4 independent experiments. **C:** Representative CARP immunoblot of NRVMs transfected with nonsilencing control or GATA4-siRNA for 24 or 48 h. **D:** Representative immunofluorescent images of NRVMs transfected with nonsilencing control or GATA4-siRNA for 48 h. NRVMs were co-stained for myomesin (green), filamentous actin (red), and DAPI (blue).

**Figure 10:**
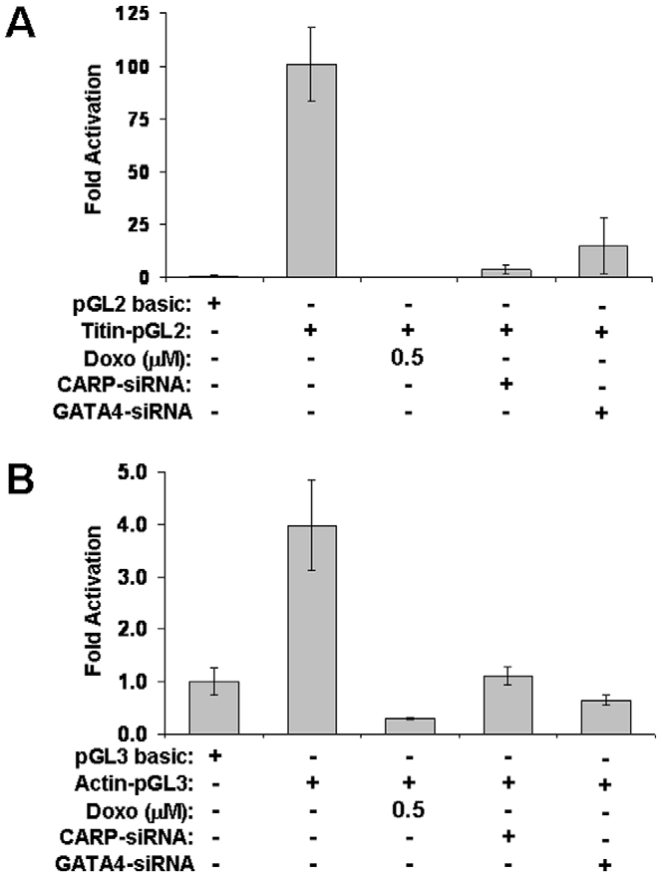
CARP and GATA4 regulate titin and actin transcription. NRVMs were cotransfected with either a titin promoter reporter (**A**) or actin promoter (**B**) along with 0.5 µM doxorubicin, CARP-siRNA, or GATA4-siRNA. Luciferase-reporter experiments were performed in triplicate. Values were normalized to untreated pGL3 basic and shown as mean±SD from 4 independent experiments.

Previous studies have shown that doxorubicin suppresses the transcription factor GATA4 and that GATA4 regulates CARP expression [20], [22], [23]. We confirmed that 24 h of doxorubicin treatment in NRVMs resulted in depleted GATA4 levels (**Figure 8A**). Moreover, transient cotransfection experiments in HEK-293 cells showed a dose-dependent increase in CARP promoter activity with increasing concentrations of GATA4 expression vector (**Figure 9A**). There was endogenous activation of the CARP promoter in the absence of GATA4 expression vector, however, we were unable to detect GATA4 by western blot in HEK-293 cells. To further examine the role of GATA4 and CARP we performed cotransfection experiments in cardiomyocytes. Knockdown of GATA4 by siRNA markedly suppressed CARP promoter activity (**Figure 9B**) and CARP protein levels (24 h = 55±13%, 48 h = 25±17% vs. nonsilencing control; *P*<0.05, **Figure 9C**) and induced extensive cardiomyocyte sarcomere disarray (**Figure 9D**). Interestingly, CARP promoter activity was inversely related to CARP expression (**Figure 9B**), suggesting an autoregulatory negative feedback mechanism for CARP in cardiomyocytes. Given that both CARP- and GATA4-siRNA induce sarcomere disarray, we examined whether GATA4 and CARP regulate sarcomere gene transcription. Cotransfection of either a titin promoter reporter or actin promoter reporter with either 0.5 µM doxorubicin, CARP-siRNA, or GATA4-siRNA showed significant decreases in promoter activity (**Figure 10**).

Thus, GATA4 regulates CARP expression and works with CARP to co-regulate sarcomere gene expression.

### GATA4 overexpression modestly restores CARP and partially rescues the doxorubicin-induced sarcomere disarray

**Figure 11:**
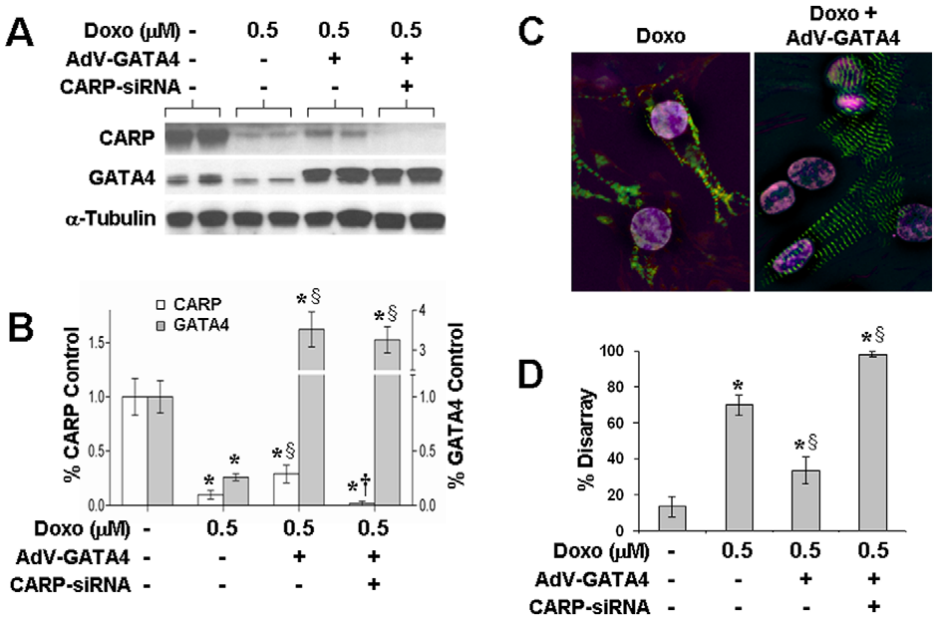
GATA4 overexpression in NRVM results in partial rescue of doxorubicin-induced sarcomere disarray. **A:** Representative immunoblots for CARP and GATA4 from NRVMs infected with AdV-GATA4 and treated with doxorubicin in the presence or absence of CARP-siRNA. **B:** Corresponding densitometry values normalized to control are shown for CARP (open bars) and GATA4 (filled bars). Shown are mean±SD, n = 6, *P*<0.05 relative to control (*), Doxo only (**§**), and Doxo+AdV-GATA4 (**†**) **C:** Immunofluorescent images of NRVMs treated with 0.5 µM doxorubicin alone or infected with AdV-GATA4 for 24 h followed by doxorubicin for 24 h. NRVMs were stained for myomesin (green), filamentous actin (red), and DAPI (blue). **D:** Bar graph shows % sarcomere disarray (n = 5–8, ∼150 cells counted per experiment). Values shown as mean±SD, *P*<0.05 relative to control (*) and Doxo only treatment (**§**).

Since GATA4 and CARP regulate sarcomere gene expression and GATA4 is upstream of CARP, we examined whether GATA4 overexpression could rescue the doxorobucin-induced sarcomere disarray phenotype. NRVMs treated with 0.5 µM doxorubicin showed significant depletion of GATA4 and CARP levels (**Figures 11A and B**). When infected with AdV-GATA4 for 24 h prior to doxorubicin, GATA4 levels were increased and CARP levels were modestly but significantly higher when compared to doxorubicin treatment alone. Overexpression of GATA4 attenuated the doxorubicin-induced sarcomere disarray as evidenced by preservation of striated M-line immunostaining (**Figures 11C and D**). Having modest levels of CARP with GATA4 overexpression appears to be critical as depletion of CARP by siRNA cotransfection abolished the AdV-GATA4 rescue effect in doxorubicin treated cells, resulting in complete sarcomere disarray in almost all cells examined (**Figures 11B and D**). Thus, GATA4 overexpression can partially rescue the doxorubicin–induced sarcomere disarray phenotype, and both GATA4 and CARP are required for maintaining cardiac sarcomeres.

## Discussion

Even though CARP was first identified as an exquisitely sensitive target of doxorubicin [8], studies examining the relationship between CARP and doxorubicin cardiotoxicity have been scant. In the present work we show that doxorubicin depletes both GATA4 and CARP levels in cultured cardiomyocytes, and that GATA4 overexpression (but not CARP overexpression) was able to attenuate doxorubicin-induced sarcomere disarray. We demonstrate that GATA4 directly regulates CARP and that the protective effect of GATA4 was mediated in part by modulating CARP expression and downstream sarcomere genes. These results are the first to identify CARP as a mediator for GATA4 in a signaling axis that converges to control sarcomere gene expression and maintain organized sarcomeres.

Doxorubicin induces sarcomere disarray and a rapid decrease in CARP protein levels in both ARVM and NRVM, with the phenotype appearing to be more severe in NRVM. CARP is believed to be a “hot spot”, titin-based, mechanosensory unit as it interacts with the elastic N2A domain of titin (for reviews see [28], [29]). Consistent with our previous report, doxorubicin induced titin degradation in ARVMs and co-treatment with a calpain inhibitor restored titin levels and preserved myofibrillar structure [24]. However, calpain inhibition did not preserve CARP levels suggesting that 1) CARP is not a calpain substrate and 2) the susceptibility of CARP to doxorubicin is independent of a preserved titin and/or sarcomere structure. The latter might be explained by impaired CARP binding to N2A titin, perhaps due to post-translational modifications, leading to enhanced degradation of free CARP by some other proteolytic mechanism. However, in this study ARVMs treated with cycloheximide in the presence or absence of doxorubicin showed no difference in the rate of CARP loss, thus arguing against accelerated CARP degradation; instead we were able to demonstrate that doxorubicin induced transcriptional inhibition of CARP. Support for this comes from a previous study showing doxorubicin-induced suppression of CARP transcription via activation of a H7-sensitive serine/threonine kinase pathway [30].

Neonatal cardiomyocytes subjected to stretch accumulate CARP in the sarcomeric I-band as well as in the nucleus, suggesting that CARP might couple mechanical strain to muscle gene transcription [6]. Following doxorubicin exposure, we also observed a transient sarcomeric to nuclear translocation of CARP. We speculate that in response to doxorubicin-induced mechanical perturbation (titin degradation) sarcomeric CARP localizes to the nucleus to modulate myofilament gene transcription; this gene transcription ultimately ceases as total CARP levels are eventually depleted with doxorubicin. It is unclear how CARP regulates cardiac gene expression, but CARP is known to be a transcriptional co-factor and has been shown to interact *in vitro* with multiple transcription factors involved in cardiac gene expression, including YB-1, HAND2, and HEY1 [9], [31].

The cardiac transcription factor GATA4 plays a pivotal role in cardiomyocyte hypertrophy and survival and it has been implicated in sarcomere gene transcription and regulation of the sarcomere assembly process [18], [32]. These diverse processes are likely mediated by different downstream effectors, which remain poorly defined. GATA4 is known to be sensitive to doxorubicin [19], [20], [22], [23]. Here we show that selective siRNA knockdown of GATA4 suppressed CARP promoter activity, depleted CARP protein levels, and induced extensive cardiomyocyte sarcomere disarray. These findings suggest that doxorubicin-induced depletion of GATA4 is directly responsible for loss of CARP, and they implicate CARP as a downstream mediator of GATA4 in regulating sarcomere maintenance. Overexpression of GATA4 enhanced CARP promoter activity in HEK293 cells whereas GATA4 siRNA knockdown in cardiomyocytes resulted in suppression of CARP promoter activity, confirming that GATA4 directly regulates CARP. CARP siRNA knockdown induced marked cardiomyocyte sarcomere disarray as seen with GATA4 siRNA, and either CARP or GATA4 siRNA resulted in significant attenuation in promoter activity of sarcomere genes, titin and α-actin. Overexpression of CARP failed to rescue the doxorubicin-induced sarcomere disarray phenotype, which was not unexpected as the concomitant loss of GATA4 is predicted to inhibit other downstream pathways distinct from sarcomeric organization such as survival pathways [19]. Furthermore, CARP is hypothesized to be a mechanosensor linking changes in titin mechanical strain to gene transcription, and it is unclear how overexpression of ectopic CARP might affect this endogenous mechanosensing or transcriptional activity of CARP. It is interesting that CARP promoter activity was inversely related to CARP expression, suggesting a negative feedback mechanism to strictly control CARP levels in cardiomyocytes. As previously noted, CARP is a cofactor for multiple cardiac transcription factors and stringent regulation of CARP is perhaps necessary to limit an exaggerated hypertrophic response. Future studies are needed to examine the role of CARP in cardiac hypertrophy.

Overexpression of GATA4, on the other hand, markedly attenuated doxorubicin-induced cardiomyocyte sarcomere disarray. This finding is consistent with previous studies which showed that GATA4 overexpression protects against cardiomyocyte apoptosis and autophagy due to doxorubicin toxicity [19], [20], [21]. These previous studies indicated that GATA4 upregulates antiapoptotic factors Bcl-X and Bcl2, and Bcl2 in turn suppresses autophagy-related genes [19], [21]. Interestingly, GATA4 overexpression modestly, but significantly, increased CARP levels in doxorubicin treated cardiomyocytes. Our finding that knockdown of this modest increase in CARP completely abrogated the rescue of GATA4 overexpression in doxorubicin treated cells suggest that CARP and GATA4 are both essential in maintaining organized sarcomeres. Our finding that CARP plays a central role in sarcomere maintenance in cultured cardiomyocytes, appears to contradict a study by Barash et al. which showed a relatively mild skeletal muscle phenotype in a triple KO mouse model of the MARP genes (including CARP), with no overt cardiac phenotype reported [17], [33]. One possible explanation is that other titin-based mechanosensors can compensate for the lack of CARP *in vivo*, e.g the MLP/telethonin/N-titin, MURF2/titin kinase, or FHL1/N2B-titin complexes [34], [35], [36]. The possibility that CARP is dispensable for basal *in vivo* cardiac function but is important under conditions of stress, such as pressure overload or myocardial infarction, requires further investigation. We also cannot rule out the possibility that when cultured *in vitro*, cardiomyocytes remodel and adapt to the rigid two-dimensional environment, where CARP becomes absolutely essential for sarcomere integrity.

GATA4 and the cardiac-enriched transcription factor Nkx2.5 are known mutual co-activators [37], and a previous study has shown that Nkx2.5 and GATA4 cooperatively regulate CARP expression [22], [37]. The GATA4/Nkx2.5 interaction has been shown to mediate a mechanical stretch-activated hypertrophic program in cardiomyocytes that enhances sarcomere assembly and organization [38]. We speculate that Nkx2.5, GATA4, and CARP all converge on a final common signaling pathway to regulate sarcomere gene transcription and sarcomere maintenance, and that loss of any one of these factors results in cardiomyocyte sarcomere disarray. We further speculate that cardiomyocyte mechanical stretch targets this same Nkx2.5/GATA4/CARP signaling pathway to induce cardiomyocyte hypertrophy and enhance sarcomere organization and assembly. A recent study showed that Ankrd2, another member of the MARP family of proteins enriched in skeletal muscle, interacts with transcriptional regulators and structural and signaling proteins to affect a multitude of pathways including myogenesis, gene expression, as well as intra- and intercellular signaling [39]. We anticipate that future studies on CARP will uncover novel signaling pathways and processes with important regulatory functions in the heart.

**Figure 12:**
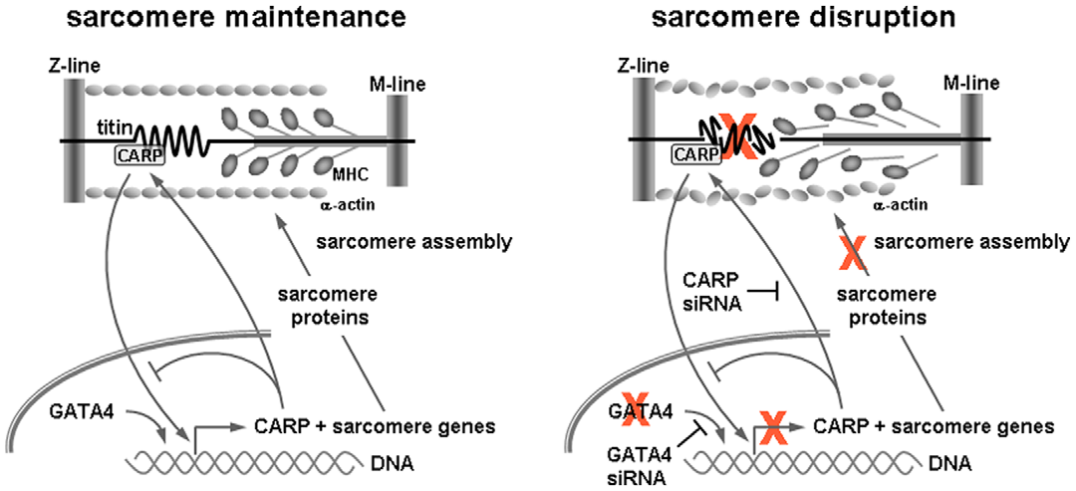
Cardiac sarcomere structure is maintained by a balance between sarcomere protein synthesis and degradation. We postulate that GATA4 regulates CARP expression and both act to induce sarcomere gene transcription. CARP expression is subject to a negative feedback regulatory mechanism. GATA4 siRNA and CARP siRNA both inhibit CARP signaling and sarcomere gene expression, resulting in an imbalance in protein turnover and eventual sarcomere disruption. Doxorubicin (actions marked by red x in figure) exacerbates sarcomere disruption by downregulation of GATA4 and CARP, degrading key sarcomeric proteins (e.g. titin), and inhibiting new sarcomere protein synthesis via transcriptional/translational impairment.

The mechanisms of anthracycline cardiotoxicity are diverse and include 1) oxidative stress and membrane lipid damage, 2) calcium overload, and 3) inhibition of protein transcription and translation (reviewed in [40], [41]). These conditions collectively lead to myofibrillar disarray, where acceleration of myofilament protein degradation and simultaneous repression of muscle gene expression leads to a net negative balance of sarcomeric proteins. In this paradigm, the downregulation of GATA4 and CARP contributes in part to this phenotype by preventing synthesis of sarcomere proteins and sarcomere assembly (**Figure 12**). The hypothesis that CARP/N2A-titin make up a stretch-sensing unit that intersects with Nkx2.5/GATA4 signaling is particularly attractive as a mechanism for cardiomyocytes to rapidly adjust sarcomere homeostasis to the changing physiological demands of the heart.

## Supporting Information

Figure S1 **CARP expression in Adult Rat Ventricular Myocyte (ARVM).** **A:** Total mRNA was isolated from ARVM and CARP expression was detected by RT-PCR using primers that targeted a 197 bp portion of CARP cDNA. RT-PCR was performed at various RNA concentrations and PCR products were run on 10% agarose gel and visualized with ethidium bromide. **B:** Total protein was extracted from ARVM and lysates were subjected to western blot using purified anti-CARP antibody.
(TIF)

Figure S2 **High magnification images of α-actinin (red) and CARP (green) and shown below is the corresponding fluorescence intensity along the white arrows in the images.** The dashed lines in the histograms are aligned with a peak in each channel, indicating labeling of CARP on either side of the Z-line.
(TIF)

Figure S3 **ARVMs were treated with 1 µM doxorubicin or transfected with 50 nM CARP siRNA at various time points.** Cells were fixed and immunostained for CARP (green) and myomesin (blue).
(TIF)

Figure S4 **Time-lapse fluorescence microscopy of CARP siRNA induced myofibrillar disarray.** ARVMs were infected for 24 hours with an adenoviral construct containing a truncated N-terminal epitope of myomesin fused to GFP which localizes to the M-line. Following M-line fluorescence expression, time-lapse microscopy was performed on ARVMs that were untreated (A, B, C), treated with nonsilencing siRNA (D, E, F), or transfected with CARP siRNA (G, H, I). Images were taken at 0, 2, and 4 days (d0, d2, d4) for untreated and nonsilencing siRNA, and at 0, 1, and 2 days (d0, d1, d2) for CARP siRNA.
(TIF)

## References

[1] Singal PK, Deally CM, Weinberg LE (1987). Subcellular effects of adriamycin in the heart: a concise review.. J Mol Cell Cardiol.

[2] Singal PK, Li T, Kumar D, Danelisen I, Iliskovic N (2000). Adriamycin-induced heart failure: mechanism and modulation.. Mol Cell Biochem.

[3] Coumans JV, Yeoh T, Seeto RK, Keogh A, Brennan K (1997). Variations in the relative mRNA levels of actins and myosin heavy chains do not produce corresponding differences in their proteins in the adult human heart.. J Mol Cell Cardiol.

[4] Ikeda K, Emoto N, Matsuo M, Yokoyama M (2003). Molecular identification and characterization of a novel nuclear protein whose expression is up-regulated in insulin-resistant animals.. J Biol Chem.

[5] Kemp TJ, Sadusky TJ, Saltisi F, Carey N, Moss J (2000). Identification of Ankrd2, a novel skeletal muscle gene coding for a stretch-responsive ankyrin-repeat protein.. Genomics.

[6] Miller MK, Bang ML, Witt CC, Labeit D, Trombitas C (2003). The muscle ankyrin repeat proteins: CARP, ankrd2/Arpp and DARP as a family of titin filament-based stress response molecules.. J Mol Biol.

[7] Chu W, Burns DK, Swerlick RA, Presky DH (1995). Identification and characterization of a novel cytokine-inducible nuclear protein from human endothelial cells.. J Biol Chem.

[8] Jeyaseelan R, Poizat C, Baker RK, Abdishoo S, Isterabadi LB (1997). A novel cardiac-restricted target for doxorubicin. CARP, a nuclear modulator of gene expression in cardiac progenitor cells and cardiomyocytes.. J Biol Chem.

[9] Zou Y, Evans S, Chen J, Kuo HC, Harvey RP (1997). CARP, a cardiac ankyrin repeat protein, is downstream in the Nkx2-5 homeobox gene pathway.. Development.

[10] Aihara Y, Kurabayashi M, Saito Y, Ohyama Y, Tanaka T (2000). Cardiac ankyrin repeat protein is a novel marker of cardiac hypertrophy: role of M-CAT element within the promoter.. Hypertension.

[11] Bang ML, Mudry RE, McElhinny AS, Trombitas K, Geach AJ (2001). Myopalladin, a novel 145-kilodalton sarcomeric protein with multiple roles in Z-disc and I-band protein assemblies.. J Cell Biol.

[12] Torrado M, Nespereira B, Lopez E, Centeno A, Castro-Beiras A (2005). ANKRD1 specifically binds CASQ2 in heart extracts and both proteins are co-enriched in piglet cardiac Purkinje cells.. J Mol Cell Cardiol.

[13] Witt SH, Labeit D, Granzier H, Labeit S, Witt CC (2005). Dimerization of the cardiac ankyrin protein CARP: implications for MARP titin-based signaling.. J Muscle Res Cell Motil.

[14] Witt CC, Witt SH, Lerche S, Labeit D, Back W (2008). Cooperative control of striated muscle mass and metabolism by MuRF1 and MuRF2.. Embo J.

[15] Arimura T, Bos JM, Sato A, Kubo T, Okamoto H (2009). Cardiac ankyrin repeat protein gene (ANKRD1) mutations in hypertrophic cardiomyopathy.. J Am Coll Cardiol.

[16] Duboscq-Bidot L, Charron P, Ruppert V, Fauchier L, Richter A (2009). Mutations in the ANKRD1 gene encoding CARP are responsible for human dilated cardiomyopathy.. Eur Heart J.

[17] Mikhailov AT, Torrado M (2008). The enigmatic role of the ankyrin repeat domain 1 gene in heart development and disease.. Int J Dev Biol.

[18] Oka T, Maillet M, Watt AJ, Schwartz RJ, Aronow BJ (2006). Cardiac-specific deletion of Gata4 reveals its requirement for hypertrophy, compensation, and myocyte viability.. Circ Res.

[19] Aries A, Paradis P, Lefebvre C, Schwartz RJ, Nemer M (2004). Essential role of GATA-4 in cell survival and drug-induced cardiotoxicity.. Proc Natl Acad Sci U S A.

[20] Kim Y, Ma AG, Kitta K, Fitch SN, Ikeda T (2003). Anthracycline-induced suppression of GATA-4 transcription factor: implication in the regulation of cardiac myocyte apoptosis.. Mol Pharmacol.

[21] Kobayashi S, Volden P, Timm D, Mao K, Xu X (2010). Transcription factor GATA4 inhibits doxorubicin-induced autophagy and cardiomyocyte death.. J Biol Chem.

[22] Kuo H, Chen J, Ruiz-Lozano P, Zou Y, Nemer M (1999). Control of segmental expression of the cardiac-restricted ankyrin repeat protein gene by distinct regulatory pathways in murine cardiogenesis.. Development.

[23] Maeda T, Sepulveda J, Chen HH, Stewart AF (2002). Alpha(1)-adrenergic activation of the cardiac ankyrin repeat protein gene in cardiac myocytes.. Gene.

[24] Lim CC, Zuppinger C, Guo X, Kuster GM, Helmes M (2004). Anthracyclines induce calpain-dependent titin proteolysis and necrosis in cardiomyocytes.. J Biol Chem.

[25] Shi Y, Reitmaier B, Regenbogen J, Slowey RM, Opalenik SR (2005). CARP, a cardiac ankyrin repeat protein, is up-regulated during wound healing and induces angiogenesis in experimental granulation tissue.. Am J Pathol.

[26] Warren CM, Krzesinski PR, Greaser ML (2003). Vertical agarose gel electrophoresis and electroblotting of high-molecular-weight proteins.. Electrophoresis.

[27] Sawyer DB, Zuppinger C, Miller TA, Eppenberger HM, Suter TM (2002). Modulation of anthracycline-induced myofibrillar disarray in rat ventricular myocytes by neuregulin-1beta and anti-erbB2: potential mechanism for trastuzumab-induced cardiotoxicity.. Circulation.

[28] Granzier HL, Labeit S (2004). The giant protein titin: a major player in myocardial mechanics, signaling, and disease.. Circ Res.

[29] Linke WA (2008). Sense and stretchability: the role of titin and titin-associated proteins in myocardial stress-sensing and mechanical dysfunction.. Cardiovasc Res.

[30] Aihara Y, Kurabayashi M, Tanaka T, Takeda SI, Tomaru K (2000). Doxorubicin represses CARP gene transcription through the generation of oxidative stress in neonatal rat cardiac myocytes: possible role of serine/threonine kinase-dependent pathways.. J Mol Cell Cardiol.

[31] Kojic S, Nestorovic A, Rakicevic L, Belgrano A, Stankovic M (2010). A novel role for cardiac ankyrin repeat protein Ankrd1/CARP as a co-activator of the p53 tumor suppressor protein.. Arch Biochem Biophys.

[32] Charron F, Tsimiklis G, Arcand M, Robitaille L, Liang Q (2001). Tissue-specific GATA factors are transcriptional effectors of the small GTPase RhoA.. Genes Dev.

[33] Barash IA, Bang ML, Mathew L, Greaser ML, Chen J (2007). Structural and regulatory roles of muscle ankyrin repeat protein family in skeletal muscle.. Am J Physiol Cell Physiol.

[34] Knoll R, Hoshijima M, Hoffman HM, Person V, Lorenzen-Schmidt I (2002). The cardiac mechanical stretch sensor machinery involves a Z disc complex that is defective in a subset of human dilated cardiomyopathy.. Cell.

[35] Lange S, Xiang F, Yakovenko A, Vihola A, Hackman P (2005). The kinase domain of titin controls muscle gene expression and protein turnover.. Science.

[36] Sheikh F, Raskin A, Chu PH, Lange S, Domenighetti AA (2008). An FHL1-containing complex within the cardiomyocyte sarcomere mediates hypertrophic biomechanical stress responses in mice.. J Clin Invest.

[37] Durocher D, Charron F, Warren R, Schwartz RJ, Nemer M (1997). The cardiac transcription factors Nkx2-5 and GATA-4 are mutual cofactors.. Embo J.

[38] Pikkarainen S, Tokola H, Majalahti-Palviainen T, Kerkela R, Hautala N (2003). GATA-4 is a nuclear mediator of mechanical stretch-activated hypertrophic program.. J Biol Chem.

[39] Belgrano A, Rakicevic L, Mittempergher L, Campanaro S, Martinelli VC (2011). Multi-tasking role of the mechanosensing protein Ankrd2 in the signaling network of striated muscle.. PLoS One.

[40] Chen B, Peng X, Pentassuglia L, Lim CC, Sawyer DB (2007). Molecular and cellular mechanisms of anthracycline cardiotoxicity.. Cardiovasc Toxicol.

[41] Sawyer DB, Peng X, Chen B, Pentassuglia L, Lim CC (2010). Mechanisms of anthracycline cardiac injury: can we identify strategies for cardioprotection?. Prog Cardiovasc Dis.

