# Supplementary figures and images for: Disruption of a GATA4/Ankrd1 Signaling Axis in Cardiomyocytes Leads to Sarcomere Disarray: Implications for Anthracycline Cardiomyopathy
Source: PLoS One. 2012 Apr 20;7(4):e35743. doi: 10.1371/journal.pone.0035743 (PMC3332030; doi:10.1371/journal.pone.0035743)

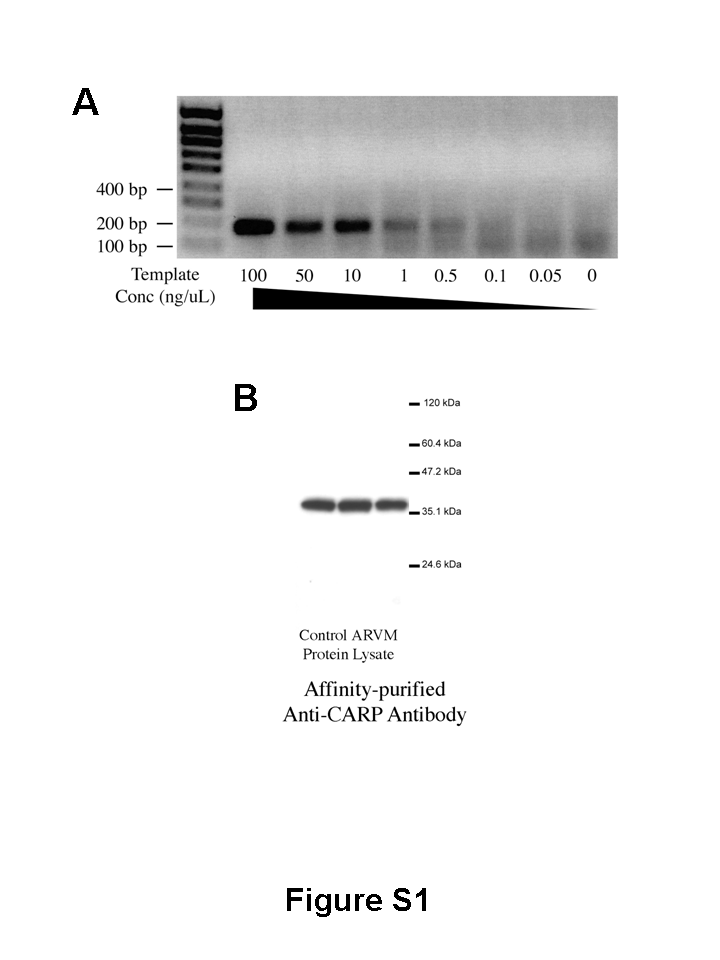

Supplement: Figure S1 — CARP expression in Adult Rat Ventricular Myocyte (ARVM). A: Total mRNA was isolated from ARVM and CARP expression was detected by RT-PCR using primers that targeted a 197 bp portion of CARP cDNA. RT-PCR was performed at various RNA concentrations and PCR products were run on 10% agarose gel and visualized with ethidium bromide. B: Total protein was extracted from ARVM and lysates were subjected to western blot using purified anti-CARP antibody. (TIF) [file pone.0035743.s001.tif]

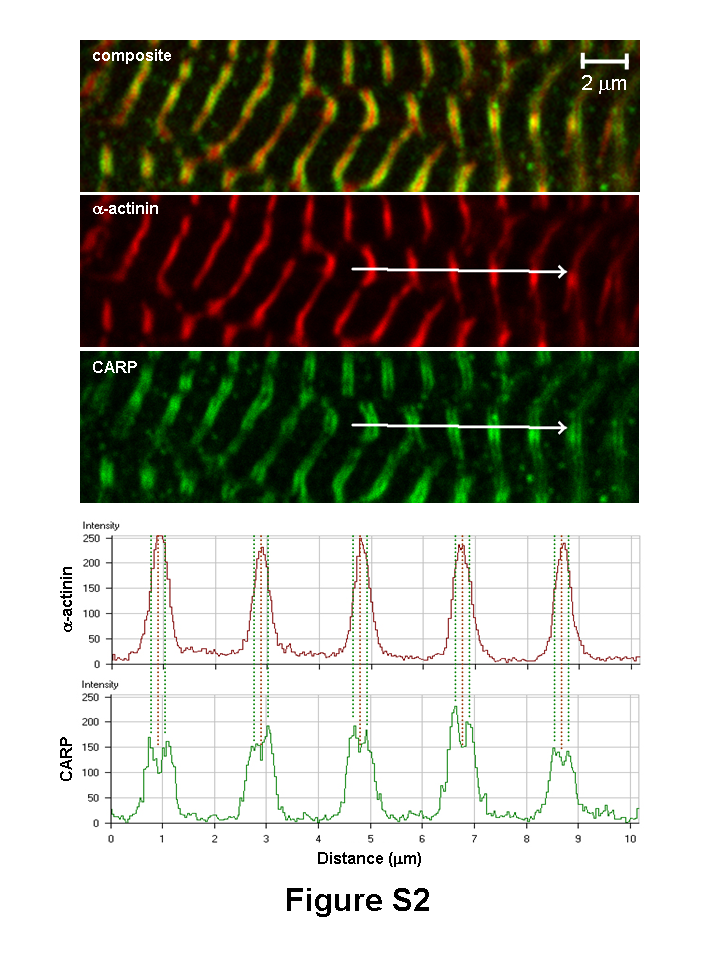

Supplement: Figure S2 — High magnification images of α-actinin (red) and CARP (green) and shown below is the corresponding fluorescence intensity along the white arrows in the images. The dashed lines in the histograms are aligned with a peak in each channel, indicating labeling of CARP on either side of the Z-line. (TIF) [file pone.0035743.s002.tif]

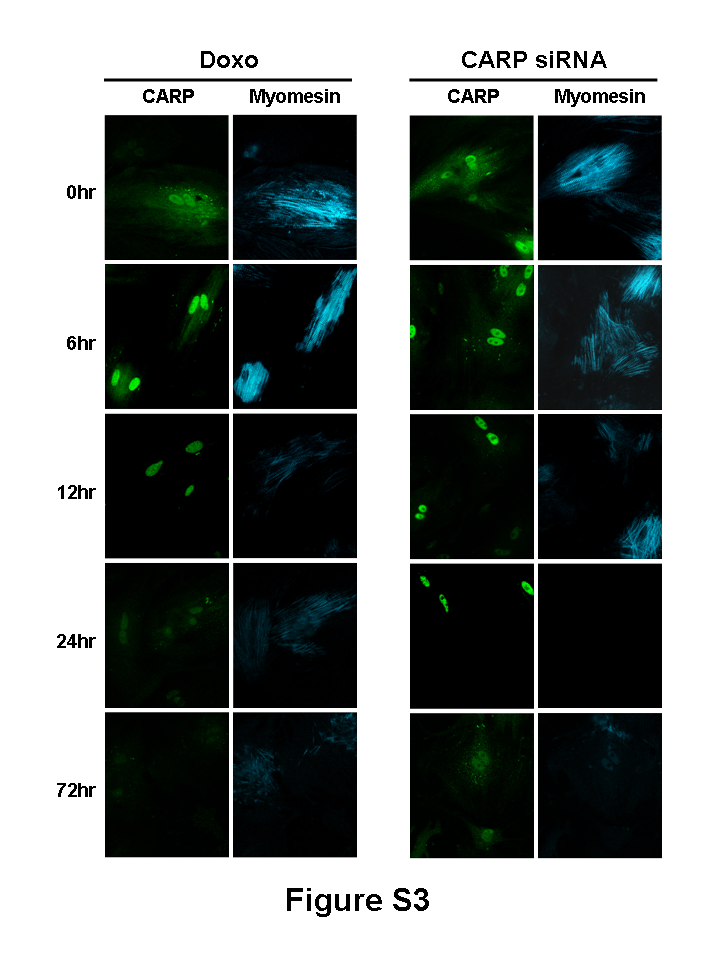

Supplement: Figure S3 — ARVMs were treated with 1 µM doxorubicin or transfected with 50 nM CARP siRNA at various time points. Cells were fixed and immunostained for CARP (green) and myomesin (blue). (TIF) [file pone.0035743.s003.tif]

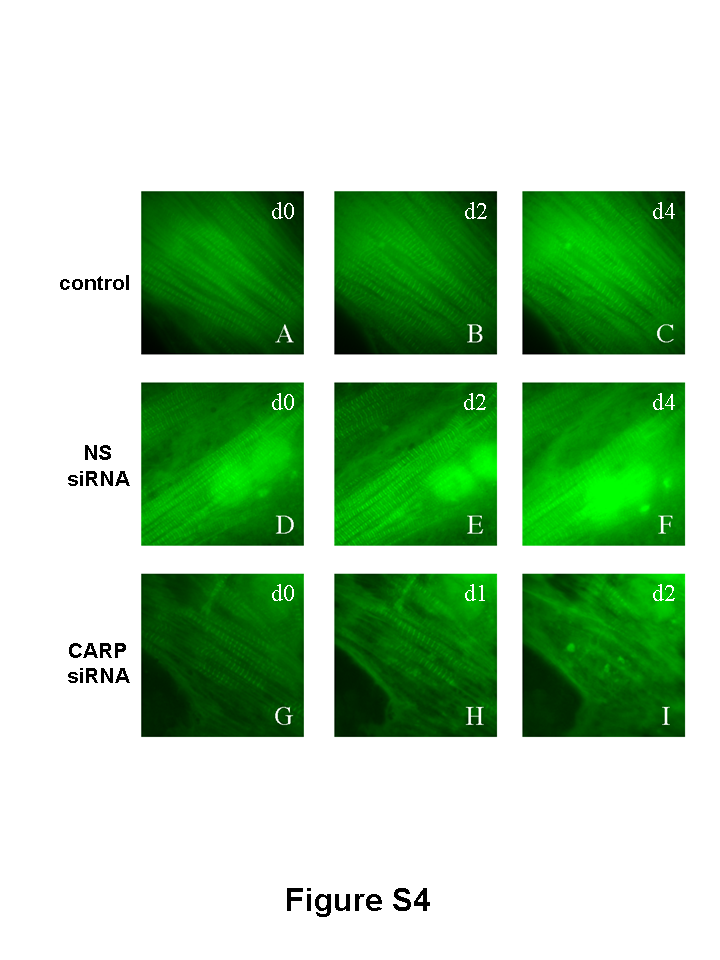

Supplement: Figure S4 — Time-lapse fluorescence microscopy of CARP siRNA induced myofibrillar disarray. ARVMs were infected for 24 hours with an adenoviral construct containing a truncated N-terminal epitope of myomesin fused to GFP which localizes to the M-line. Following M-line fluorescence expression, time-lapse microscopy was performed on ARVMs that were untreated (A, B, C), treated with nonsilencing siRNA (D, E, F), or transfected with CARP siRNA (G, H, I). Images were taken at 0, 2, and 4 days (d0, d2, d4) for untreated and nonsilencing siRNA, and at 0, 1, and 2 days (d0, d1, d2) for CARP siRNA. (TIF) [file pone.0035743.s004.tif]
